# Assessment of the priority target group of mental health service networks within a nation-wide reform of adult psychiatry in Belgium

**DOI:** 10.1186/s12913-016-1434-2

**Published:** 2016-05-17

**Authors:** Vincent Lorant, Adeline Grard, Chantal Van Audenhove, Eva Helmer, Joke Vanderhaegen, Pablo Nicaise

**Affiliations:** Institute of Health and Society (IRSS), Université catholique de Louvain, B1.30.15, Clos Chapelle-Aux-Champs, Brussels, 1200 Belgium; LUCAS (Centre for Care Research and Consultancy), Katholiek Universiteit Leuven, Minderbroedersstraat 8, postbus 5310, 3000 Leuven, Belgium

**Keywords:** Mental health policy, Mental health services, Mental health care reform, Organisational model

## Abstract

**Background:**

Belgium is currently implementing a nation-wide reform of mental health care delivery based on service networks. These networks are supposed to strengthen the community-based supply of care, reduce the resort to hospitals, and improve the continuity of care. They are also intended to supply comprehensive care to all adult mental health users. It is unclear, however, if one single model of network can target the needs of the whole adult population with mental health problems.

**Methods:**

In 2011, ten networks were commissioned and assessed. Networks included a total of 635 services of different types. Services were asked to select 10 users by systematic sampling and to state whether these users were considered as a priority for care in the network. Sociodemographic, social integration level, diagnoses, and psycho-social functioning variables were also collected.

**Results:**

Two thousand four hundred ninety users were included, and 1564 were given priority for network care. Priority was higher for men than for women (69.9 % versus 56.2 %), and for non-nationals than for Belgians (72.6 % versus 61.9 %). Users were designated priority when they had poor psycho-social functioning (HoNOS > 17, OR = 3.15, *p* < 0.001), personality disorder or schizophrenia (OR = 1.54, *p* < 0.001), and a medium level of social integration (SIX = [2,3], OR = 1.57, *p* < 0.001). Less socially integrated patients (SIX < 1, OR = 0.53, *p* < 0.001) and users of community and social services were less likely to be selected.

**Conclusion:**

Although the reform was intended for the whole population of adults with mental health problems, the users selected have a profile of severe mentally-ill users with social deprivation and poor social functioning. Policy may have been over-ambitious trying to address the whole population with one single type of service network. The actual selection process of users makes it less likely that the reform will achieve all its objectives.

## Background

Since the shift of most mental health care delivery systems towards community care, continuity of care has been the central concern of health policy-makers and service managers [1–5]. The lack of continuity of care, in particular in community-based mental health systems, led to lower levels of improvement than expected in terms of symptoms and social functioning [6–9]. Among other organisational settings, community care networks and other forms of service partnerships have been suggested in order to improve the continuity of care for patients with psychiatric disorders [5, 10, 11]. Such service networks were also the core organisational principle of the mental-health care delivery reform which has been implemented in Belgium since 2010 [12, 13].

The health care system in Belgium may be described as having three fundamental characteristics. Firstly, the health care system is organised as a regulated-market system [14, 15]. This implies extensive decision-making autonomy for users, clinicians, and providers. In practice, users have the freedom to choose clinicians and services, regardless of territorial or referral criteria. Similarly, services and health providers, which are predominantly non-for-profit publicly funded organisations, have extensive freedom of choice regarding care delivery policies and partnership agreements. By contrast, public health authorities have weak powers of regulation, with decisions being mainly made within a framework of negotiations between stakeholders and subsequently enacted by public authorities [16]. Compulsory health insurance covers most of the fee-for-service costs within this system.

Secondly, the health care system is highly fragmented. Authority in the health service provision and funding is shared between five different levels of authority, leading to a complex distribution of policy-making between the federal state and the regions. As a result, hospitals, community mental health services, and social care services depend on multiple funding schemes and are organised within different policy frameworks. This complexity hinders collaborative processes between services.

Thirdly, before the reform policy was implemented, the process of deinstitutionalisation was far from complete. In 2008, there were still 152 psychiatric beds for 100,000 inhabitants, the second-highest number in Europe [17]. Moreover, community services and hospital psychiatric beds have been unevenly distributed across the country [12, 14]. Hence, despite the development of community care services, large psychiatric hospitals with beds for patients with long-term conditions have remained the basic care supply for adult psychiatric care.

**Fig. 1:**
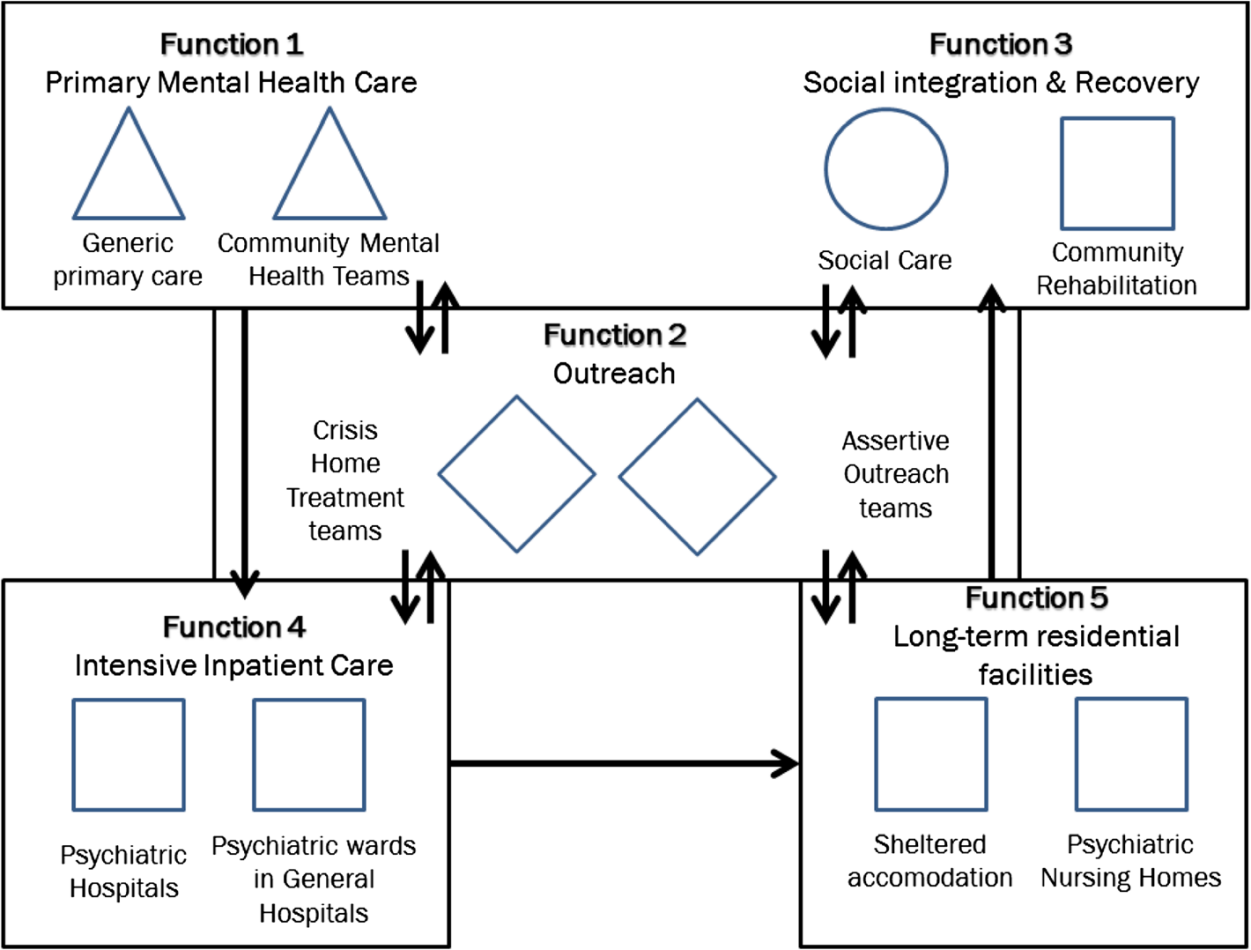
Overview of the Belgian mental health delivery system adapted from the reform blueprint [12], Belgian Mental Health Network reform 2013. Figure legend: The five basic care functionalities suggested by the reform blueprint are represented with the type of services mainly involved in the implementation of these functionalities. Triangles represent services mainly organised at the Regional level; squares represent services mainly organised at the Federal level; circles represent services mainly organised at the local level; and diamonds represent the newly established mobile teams

The mental-health care delivery reform is based on the establishment of networks of mental health services which are intended to supply circuits of comprehensive care to all adult mental health service users. The programme theory underlying this reform can be summarised as follows: the strengthening of a community-based supply of care (structure), together with mechanisms for care coordination and a reduction of the resort to psychiatric hospitals (process), should improve social rehabilitation and user recovery (outcomes). To achieve these overarching aims, networks of mental health services have been asked to supply five basic care functionalities that are supposed to cover the mental health-care needs of the whole adult population: [1] prevention, early detection, and primary care for mental health disorders, [2] outreach and crisis interventions, [3] recovery and social rehabilitation, [4] intensive residential treatment for acute cases, and [5] long-term care and housing facilities. Networks of services are based on voluntary projects at the local level and initiated by psychiatric hospitals. These networks of services aim to include all the relevant services for the mental health needs of the adult population: in- and outpatient mental health services, primary care, outreach, day care, vocational, housing, and all other social care services (see Fig. 1). The basic funding mechanism of the present reform is based on the conversion of financial means dedicated to long-term psychiatric beds into collaboration with community services [12]. The programme theory underlying this reform has been analysed in detail elsewhere [13, 18].

However, it is unclear whether such diverse types of services, within a fragmented policy context, can deliver comprehensive care for the whole adult population. Indeed, care coordination settings are supposed to share a common, operational definition of the user target group. In particular, research on health service networks has suggested that it is not possible to integrate all services for all patients [19]: service networks should develop either relationships between a small number of services for all patients (hereafter, “depth of relationships”), or relationships between a large number of services for a narrowly-defined group of patients (hereafter, “breadth of relationships”) [4, 20]. Programme evaluation theory also suggests that a clear target group is a key factor for effectiveness, contributing to tailoring intervention designs, resources allocation, process monitoring, and outcome assessment [21].

Accordingly, the aim of this study was to analyse whether the newly established service networks are actually targeting the whole population of adults with mental-health care needs, or whether service networks have developed with a more specific group of users in mind. In the latter event, the study would set out to describe the characteristics of this group and how those characteristics affect service networks.

## Method

### Study design and data collection

**Fig. 2:**
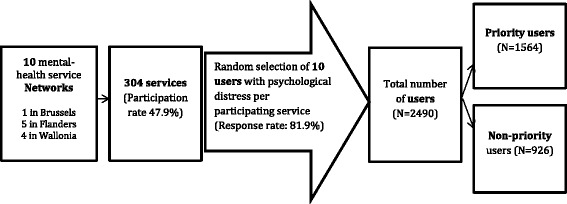
Selection and inclusion of patients, Belgian Mental Health Network reform 2013

We carried out an observational study to assess which patients’ sociodemographic and clinical variables were associated with being given priority for care in the network by clinicians. At the time of the study (2013), ten mental health service networks for adults with mental health problems were established, covering urban and rural areas across the country. All networks were included in the study. The individual networks included between 14 and 140 different services, with a total of 635, and all services were invited to participate. Services were asked to select ten users by systematic sampling. The inclusion criteria were: being adult (between 18 and 65 years of age), being in contact with the service during the data collection period, and having psychological distress. No specific diagnosis was required. All included patients gave a written informed consent for participation. Clinicians of the services, knowing the users, were asked to complete an online questionnaire about the sociodemographic and clinical characteristics of each user selected (see Fig. 2). In addition, clinicians were requested to state whether, yes or no, the patient was, at that time, considered as a priority for care within the network. The wording of the question was: “Is this patient a priority for the care circuit?” Criteria for defining the priority were deliberately left undefined in order to assess how the priority group was constructed. The priority status of users had to be discussed during a staff meeting involving clinicians and health service managers in each individual service. Staff decision determined the inclusion of users into the priority group.

### Measurements

The dependent variable was “inclusion in the priority group for care in the network (yes/no)”. We assessed the association between the inclusion of the user in the priority group and the user’s social and clinical vulnerability. In particular, the level of social integration was measured using a SIX score [22]. The SIX is an index that measures the social integration of long-term psychiatric adult patients in terms of employment, housing, and social relationships. Clinical information included DSM-IV diagnoses (when available), and the Health of the Nation Outcome Scale (HoNOS), which captures the severity of psychosocial malfunctioning [23].

Additional information about the type of service and network characteristics was also included. On the one hand, previous research suggested that some types of services have a broader spectrum of user profiles, which may affect their capacity to prioritise [24]. Hence, services were classified in five groups, following the functional model of the reform: [1] primary care and community mental health services, [2] crisis resolution and assertive outreach teams, [3] rehabilitation and social care services, [4] psychiatric hospitals and psychiatric wards of general hospitals, [5] sheltered accommodation services for psychiatric patients. On the other hand, some network structures may also be best adapted for some users’ specific profiles [19]. Thus, networks were classified according to their size (number of services included) and the type of services included. Four small networks included up to 41 services; four medium networks included between 54 and 75 services; and two large networks included more than 100 services. Regarding service types, two networks included a majority of primary care and community mental health services (hereafter “primary care-centred”), five networks included a majority of rehabilitation and social care services (hereafter “social care-centred”), and three networks included a majority of psychiatric hospital wards (hereafter “hospital-centred”).

### Data analysis

Tabulations and chi-square tests described how the inclusion of users in the priority group was associated with users’ sociodemographic and clinical characteristics, as well as with service and network types. Patients were also compared across network types. Then, nested logistic regressions were used to analyse which covariates were associated with users’ priority status. All statistical analysis was performed using SAS 3.4 for Scientific Linux.

## Results

As shown in Fig. 1, 304 services out of the 635 actually participated in the survey (48 %), and recruited a total of 2,490 users. Table 1 describes the sample (columns 1 and 2) and the association between covariates and priority (column 3). On average, users were 43.7 (std = 13.6) years of age. Overall, 62.8 % of them were assigned priority for care within the network. Priority was higher for men than for women (69.9 % versus 56.2 %), and for non-nationals than for Belgians (72.6 % versus 61.9 %). There was an inverted U association between social integration and priority: users with a very low or a very high social integration score were less considered to be priority than users with an intermediate social integration score. The group of users designated as priority users was composed of patients with schizophrenia, psychotic, and personality disorders, and users with high HoNOS scores. Users recruited in crisis resolution and assertive outreach teams were more frequently assigned priority.

**Table 1:** Socio-demographic and clinical characteristics of patients by priority status for care within the network, Belgium, Mental Health Network reform 2013, (*n* = 2490)

Covariate	%	*N*	Priority status for care within the network (%)	Chi-square
Sex				49.8**
Female	51.7	1288	56.2	
Male	48.3	1202	69.9	
Nationality				9.9*
Foreign	8.8	219	72.6	
Belgian	91.2	2271	61.9	
Education				1.1
Low (= < lower secondary school)	48.6	1210	70.0	
High (> = Upper secondary school)	39.4	980	68.0	
Social integration Score (SIX) (/6)				141.9**
= <1	39.1	973	48.6	
2–3	39.9	994	73.7	
> =4	21.0	523	68.5	
Diagnosis				41.5**
Mental disability	2.6	65	53.8	
Schizophrenia and psychotic disorders	30.3	755	74.8	
Anxiety disorders	3.1	76	53.9	
Mood disorders	15.5	385	64.4	
Personality disorders	9.8	245	74.7	
Drug-related disorders	12.5	312	63.1	
Other	9.1	227	73.1	
HoNOS score (/48)				115.7**
= <4	39.7	989	50.7	
5–11	21.6	539	66.0	
12–17	18.6	462	70.3	
> 17	20.1	500	76.4	
Type of service				252.1**
Psychiatric wards and hospitals	37.5	933	67.7	
Sheltered accommodation services	8.2	204	71.1	
Primary care and community mental health services	18.6	462	62.3	
Rehabilitation and social care services	18.5	461	34.5	
Crisis resolution and assertive outreach teams	11.8	293	87.7	
Network orientation				16.3**
Hospital-centred	22.6	563	67.3	
Primary care-centred	12.1	301	61.8	
Social care-centred	61.9	1542	62.3	
Number of services in the network				27.5**
Small	28.6	713	62.6	
Medium	42.0	1046	60.1	
Large	26.0	647	69.7	
Priority status for care within the network				
No priority	37.2	926		
Priority	62.8	1564		

*p < 0.01

**p <0.001

Table 2 shows patients’ characteristics according to network types. Standard deviations were quite similar across network types. However, hospital-centred networks recruited more frequently male patients, with a lower social integration score (SIX) and lower psychosocial functioning (higher HoNOS score) compared with the two other types of networks. Conversely, social care-centred networks recruited patients with a better score of social integration and psychosocial functioning. The difference was particularly marked for social integration: 53 % of patients from the hospital-centred networks had a very low social integration score compared with 32 % of the patients from the social care-centred networks. These differences are likely to bear on priority assignation.

**Table 2:** Socio-demographic, social integration, and psychosocial functioning scores according to network-orientation type, Belgium, Mental Health Network reform 2013, (*n* = 2490)

	Network orientation						
	Hospital-centered		Primary care-centred		Social care-centred		F-test / Chi-square^a^
	Mean	Std	Mean	Std	Mean	Std	
Age (y)	43.2	14.6	45.8	12.6	43.5	13.3	2.8 (0.04)
Male (%)	55.6	49.7	38.5	48.7	48.9	50.0	45.9 (<0.01)
SIX score = <1 (%)	52.8	50.0	38.9	48.8	32.0	46.7	128.1 (<0.01)
HoNOS (score)	15.3	7.4	14.4	6.8	13.8	7.5	4.0 (0.01)

^a^F-test for age and HoNOS score, chi-square test for sex and SIX score; *p* value in brackets

Table 3 describes the association between priority and patient and service characteristics by means of logistic regressions. Model 1 examines, separately, logistical relations between each covariate and the priority status of users. The typical user deemed to be a priority for care within networks is a male foreigner, with severe symptoms of schizophrenia or personality disorders, and with an intermediate level of social integration, selected by crisis resolution and assertive outreach teams (Odds Ratio (OR) = 3.4, *p value (p)* < 0.001) within a large hospital-centred network. The likelihood of selection increased with the HoNOS score: patients with a HoNOS score of 17+ were 3.15 times more likely to be selected than patients with a HoNOS score lower than 4. Model 2, which considers users’ characteristics, gave similar results: users with schizophrenia and users with a high HoNOS score were more likely to be assigned priority, while users with a very low social integration score were less likely to be selected. Model 3, which added network and service characteristics to the design of Model 2, led to an important improvement in the model fit (Wald Chi-square moving from 99.9 (Model 2) to 152.3 (Model 3), *p* < 0.0001): crisis resolution and assertive outreach teams, on the one hand, and hospital-centred networks, on the other hand, were more likely to designate patients as priority cases.

**Table 3:** Priority status for care within the network by socio-demographic and clinical characteristics, Belgium, Mental Health Network Reform 2013: results of the logistic regressions (*n* = 2490)

Covariates	Model 1^(2)^		Model 2^(2)^		Model 3^(2)^	
	OR	95 % CI	OR	95 % CI	OR	95 % CI
Sex (Ref. male)						
Female	0.74***	(0.68–0.81)	0.97	(0.79–1.19)	0.92	(0.74–1.14)
Age	0.99***	(0.98–0.99)	0.99**	(0.98–1.00)	0.98***	(0.98–0.99)
Nationality (Ref. Foreign)						
Belgian	0.61**	(0.45–0.83)	0.76	(0.51–1.12)	0.74	(0.49–1.13)
Education Group (Ref. > Upper secondary school)						
Low (<=Lower secondary school)	0.91	(0.76–1.09)	1.24*	(1.01–1.52)	1.35**	(1.09–1.68)
Social integration Score (SIX) (Ref. > =4–6)						
< 1	0.53***	(0.47–0.59)	0.63**	(0.47–0.83)	0.62**	(0.44–0.85)
2–3	1.57***	(1.39–1.77)	1.25	(0.97–1.62)	1.26*	(0.96–1.65)
Diagnosis (Ref. Drug-related disorders)						
Mental disability	0.60*	(0.39–0.93)	0.68	(0.38–1.19)	0.56	(0.30–1.04)
Schizophrenia and psychotic disorders	1.54***	(1.28–1.85)	1.86***	(1.38–2.50)	1.58**	(1.15–2.17)
Anxiety disorders	0.61*	(0.41–0.90)	0.64	(0.38–1.08)	0.84	(0.48–1.46)
Mood disorders	0.94	(0.76–1.16)	1.07	(0.77–1.49)	1.02	(0.73–1.45)
Personality disorders	1.53**	(1.16–2.00)	1.67*	(1.13–2.46)	1.65*	(1.08–2.50)
Other	1.41*	(1.07–1.86)	1.68**	(1.14–2.48)	1.51	(1.00–2.27)
HoNOS Score (Ref. = <4/48)						
5–11	1.89***	(1.52–2.36)	1.05	(0.81–1.36)	1.02	(0.76–1.36)
12–17	2.31***	(1.83–2.92)	1.26	(0.96–1.66)	1.30	(0.96–1.77)
> 17	3.15***	(2.48–4.01)	1.67***	(1.27–2.21)	1.73***	(1.28–2.35)
Type of service (Ref. Psychiatric wards and hospitals)						
Sheltered accommodation services	1.17	(0.84–1.63)			1.28	(0.86–1.90)
Primary care and community mental health services	0.79*	(0.62–1.00)			0.75*	(0.56–1.00)
Rehabilitation and social care services	0.25***	(0.20–0.32)			1.05	(0.65–1.70)
Crisis resolution and assertive outreach teams	3.40***	(2.34–4.95)			2.85***	(1.90–4.28)
Network orientation (Ref. Hospital-centred care)						
Primary care centred	0.79	(0.59–1.05)			0.88	(0.53–1.45)
Social care centred	0.80*	(0.65–0.98)			0.71*	(0.54–0.93)
Number of services in the network (Ref. Medium)^(1)^						
Small	1.11	(0.91–1.35)				
Large	1.53***	(1.24–1.88)				

^(1)^We did not include network size in Model 3 because of its high association with network orientation (Cramer’s V: 0.599 and 0.869; *p* < 0.0001)

^(2)^Model 1 presents bivariate association; Model 2 includes all clinical and socio-demographic variables; Model 3 adds services and network features to Model 2

*p < 0.05; **p < 0.01; ***p < 0.001

## Discussion

### Consistency with previous studies and interpretation

As mentioned in the introduction, there is a debate about whether networks should pursue depth of relationships (a higher degree of integration of care delivery between some services for all users) or breadth of relationships (a higher degree of integration of care delivery between all services for a narrowly-defined target group of patients) [4, 19, 25]. However, the reform programme supported the establishment of service networks that include all types of services for the needs of the whole adult population [12, 13]. Our analysis suggests that the policy may have been over-ambitious: the reform has targeted a specific group of users in practice: users with psychiatric and personality disorders and poor psycho-social functioning, i.e. a group related to chronic and severe mentally ill (SMI) patients. These SMI patients were designated as those who would be more likely to benefit from care delivered within service networks, it has been suggested elsewhere [26–28].

At the same time, our results suggest that different types of services address different types of user profiles. Thus, services do not share a common vision about the type of users that should be a priority for care within the service network. This may generate tensions across services and, as a result, reduce the capacity of these services to collaborate and to provide care in partnership. Such tensions may affect service networks and other care coordination schemes, and might explain, for example, the reluctance of primary care and community mental health teams to get involved in service networks [24, 29–31]. In this kind of service network, primary and community mental health care services are asked to play a key coordination role in maintaining continuity of care for SMI patients and reducing the resort to hospitals [13, 27, 28, 32]. However, they basically address a broader group of patients, which may hinder their capacity for collaboration with services specifically catering for a more severe group of patients. This coordination role is a daunting task, carrying a huge opportunity cost to clinical face-to-face activity with patients. It may also affect the patient-doctor trust through the “three-is-a-crowd” mechanism: the more additional bodies a clinician has to coordinate with, the less the patient may trust his decision-making, particularly in the context of a health care reform [33]. The feedback that we received from the social services during the data collection also confirmed this: for example, some social care services did not want to assess the mental health status of their patients, arguing either that they had no training in the use of instruments such as the HoNOS, or that they did not want to stigmatise their clients because of their mental illness.

This ambiguity is reflected in the policy framework itself. Indeed, although the reform programme is intended for the whole adult population with mental health needs, the policy blueprint of the reform used different terms and implicitly narrower definitions of the target group population [12]. Generic mental health terms (“psychological support”, “mental health”, “mental disorders”, etc.) were most frequently used to describe users and symptoms, whereas other terms related to psychiatry (“psychiatrist”, “psychiatric problems”, “mental illness”, etc.) were used to explain previous policy reform measures and to describe care supply. Thus, although the case for the new policy was made on the basis of a broad definition of mental health problems, the proposals for its actual implementation referred to more severe psychiatric disorders.

A second important finding is that users with the lowest level of social integration were not assigned priority for care in the service networks. This may appear to contradict the reform’s goal of reducing the resort to psychiatric hospitals. However, it is consistent with previous research on the deinstitutionalisation process that showed that the most severe patients were left behind [34, 35]. One possible explanation for this apparent contradiction is that the less socially integrated patients are found in sheltered accommodation and long-term residential services, and may not be perceived as eligible for care within a network of services. However, this raises an important issue and a possible key limitation to the deinstitutionalisation process that the reform is supposed to strengthen. From a system perspective, it has been suggested elsewhere that patients require different levels of care integration according to the severity of their disorders [19]: coordination of care between different services would be best adapted to patients with mild to moderate disorders, whereas the most severe patients would require fully integrated services. Our findings suggest that mental health service networks might not be an optimal solution for SMI patients with very poor social functioning.

### Limitations

Our study had some limitations. Firstly, participation rates in the survey varied between networks, with a minimum of 20 % of the expected number of patients. However, the global mean participation rate (62 %) and the size of the sample (2,490 patients in total) were sufficient to perform quantitative analyses. Nevertheless, a selection bias cannot be totally ruled out: in particular it is likely that the networks with the lower participation rates were also those striving the hardest to implement the reform, and possibly, those that had the greatest discrepancies in terms of target group definition. Secondly, psychiatric hospitals proved more likely to participate in the survey than social and primary care services, leading to a possible bias in patient recruitment. Primary care, community mental health, and social care services accounted for 37.1 % of the total sample of users, which could be an underestimation of their overall contribution to mental health care nationwide. However, this is also an indication of their actual involvement in the development of the service networks, and reflects their understanding of such networks’ implicit target group. Thirdly, the study design deliberately left priority status undefined. Hence, it is likely that different clinicians and staff may have used different standards and criteria to make their decision, as well as elements related to their local context.

Finally, the number of networks involved (ten) was too low for a robust analysis of network-level effects. Moreover, these networks had only been operating for 2 years at the time of the study. The reform process is still ongoing; new networks have been established since then and more services have been included. Accordingly, it is likely that practices and views of priority target groups will evolve over time.

## Conclusions

Although the service networks established within the Belgian nation-wide reform are supposed to target the entire adult population with mental health needs, our study found that these networks are prioritising users with an intermediate level of social integration, who have diagnoses of schizophrenia or personality disorders, and who show poor psycho-social functioning. We also found that psychiatric wards and outreach teams designated a higher proportion of their patients as having priority for care within the network compared with other types of services. The fact that services and care providers have an extensive autonomy to prioritise patients for network care, and do not follow common rules or guidelines, strengthens the significance of the results.

Our study suggests that one size of service networks does not fit all patients, and is consistent with previous research suggesting that networks of services cannot integrate all services for all users [19]. Yet, despite the somewhat over-ambitions of the Belgian mental health care delivery reform, service networks can be considered to be a tool to deliver coordinated care to users with psychiatric disorders in a deinstitutionalised environment. Our study, however, raises three concerns: firstly, patients with the lowest level of social integration, i.e. those who are most need of integrated care in the community, are not being targeted for care within the network by clinicians. This is a key issue for the reform’s prospects of success. Secondly, community mental health and primary care services have stayed somewhat on the periphery of the newly established networks, so far. Policy-makers should consider mechanisms to reinforce their involvement in the service networks. Finally, the targeting process has to be clarified within the reform process. In particular, more specific networking and collaborative mechanisms should be tailored to address different target groups and to involve services more in line with their usual user profiles. The reform process is still ongoing, as well as the evaluation process. Further research will particularly assess the role of generic primary care services, community mental health teams, and social care services in the global mental health care provision and patient targeting.

## Ethics approval

The research protocol was reviewed and approved by the “Commissie Medische Ethiek” Ethical Committee, KULeuven, Leuven, Belgium, 12 October 2012, reference number B322201215206.

## Availability of data

Data are not publicly available as they include numerous individual-level, geo-coded, and sensitive data on a very specific target group of patients with severe mental disorders.

## Abbreviations

HoNOS: Health of the Nation Outcome Scale
SMI: severe mentally ill
